# Integrated meta‐QTL and in silico transcriptome assessment pinpoint major genomic regions responsible for spike length in wheat (*Triticum aestivum* L.)

**DOI:** 10.1002/tpg2.20492

**Published:** 2024-07-31

**Authors:** Changgang Yang, Xueting Zhang, Shihong Wang, Na Liu

**Affiliations:** ^1^ Wheat Research Institute Gansu Academy of Agricultural Sciences Lanzhou China

## Abstract

Spike length (SL) is one of the major contributors to wheat yield. Uncovering major genetic regions affecting SL is an integral part of elucidating the genetic basis of wheat yield traits and goes further pivotal for marker‐assisted selection breeding. A genome‐wide meta‐quantitative trait locus (MQTL) analysis of wheat SL resulted in the refinement of 48 MQTLs using 227 initial QTLs retrieved from previous studies published over the past decades. The average confidence interval (CI) of these MQTLs amounted to a 5.16‐fold reduction compared to the mean CI of the initial QTLs. As many as 2240 putative candidate genes (CGs) were identified from the MQTL intervals using transcriptomics data in silico of wheat, of which 58 CGs were identified based on wheat–rice homology analysis. For the key CG affecting SL, a functional kompetitive allele‐specific PCR (KASP) marker, *TaPP2C*‐*3B*‐KASP, was developed to distinguish *TaPP2C*‐*3B*‐*Hap I* and *TaPP2C*‐*3B*‐*Hap II* based on the single nucleotide polymorphism at the 272 bp (A/G). The frequency of the elite allelic variation *TaPP2C*‐*3B*‐*Hap II* with high SL remained relatively stable at about 49.62% from the 1960s to 1990s. Integration of MQTL analysis and in silico transcriptome data led to a significant increase in the reliability of CGs for the genetic regulation of wheat SL, and the haplotype analysis for key CGs *TaPP2C*‐*3B* of SL provided insights into the biological function of the *TaPP2C*‐*3B* gene.

## Core Ideas

- As many as 227 initial quantitative trait loci (QTLs) related to spike length (SL) were collected to conduct the meta‐QTL (MQTL) analysis.
- The 48 MQTLs which harbored 2240 putative candidate genes (CGs) were identified.
- A functional kompetitive allele‐specific PCR (KASP) marker was developed for *TaPP2C‐3B*.
- The haplotype *TaPP2C‐3B‐Hap II* was an elite allelic variation for high SL.

**Abbreviations**
AIC: Akaike information criterion
BC: backcross
CGs: candidate genes
CI: confidence interval
DArT: Diversity Arrays Technology
DH: double haploid
GNS: grain number per spike
GO: gene ontology
KASP: kompetitive allele‐specific PCR
KEGG: Kyoto Encyclopedia of Genes and Genomes
LOD: logarithm of odds ratio score
MAS: marker‐assisted selection
MQTL: meta‐QTL
NILs: near‐isogenic lines
PCR: polymerase chain reaction
PVE: phenotypic variation explained
QTL: quantitative trait locus
RILs: recombinant inbred lines
SL: spike length
SNP: single nucleotide polymorphism
SSR: simple sequence repeat
TGW: 1000‐grain weight
TN: tiller number
TPM: transcripts per million

## 1 INTRODUCTION

Wheat (*Triticum aestivum* L.) is one of the most widely consumed cereal food crops and serves as the main energy source for approximately one‐third of the global population (P. Gupta et al., 2020), making it a prominent candidate for global food security. In the past few decades, global wheat production has greatly increased due to the widespread adoption of the “Green Revolution” gene. However, a yield ceiling has now emerged (B. Ma et al., 2023). Moreover, in the context of global climate change, growing population, and reduction in arable land, there is a major concern that the risk of a global food crisis is increasing every year (Curtis & Halford, 2014). The need for breeding high‐yielding wheat varieties is more urgent than ever (Ashraf, 2010; H. Hu & Xiong, 2014). To address these multifaceted concerns, breeders attach significant importance to improving critical yield‐related agronomic and physiological traits to enhance wheat breeding programs worldwide (Bustos et al., 2013). In addition, in the early 21st century, with the publication of the rice genome sequence, researchers have made great progress in rice genomic research, which has facilitated the continuous enhancement of the rice breeding process (Z. Chen, Ke et al., 2022). Since then, significant strides have been made in wheat genome sequencing and the availability of large germplasm resources has increased, which heralded the beginning of the “golden age” of wheat gene function research (Xiao et al., 2022).

The formation of grain yield is a complex, polygenic, and quantitative trait that is dissected into three yield parameters, including grain number per spike (GNS), effective tiller number (TN), and 1000‐grain weight (TGW). This complexity makes increasing yield progressively challenging (Nadolska‐Orczyk et al., 2017; Xiao et al., 2022; X. Xu et al., 2022). Among these three components, GNS and TGW are closely associated with spike morphology (P. Cao et al., 2019; Ji et al., 2021). Given the significance of this issue, it is crucial to delve deeper into the genetic mechanisms and developmental foundations of spike morphology. This exploration is essential not only for increasing spikelet numbers but also for enhancing the grain setting rate of spikelet (Malik et al., 2021). Moreover, a significant increase in spike length (SL), spike compactness, and fertile floret number is necessary to enhance wheat yield (Z. Guo et al., 2017). A published study shows that SL exhibited significant positive correlations with wheat yield (Donmez et al., 2001; Z. Guo, Zhao et al., 2018; M. Kumar et al., 2022; Z. Liu, Jin et al., 2023; Wu et al., 2012). Furthermore, genotypes with much longer spikes are often associated with reduced severity of Fusarium head blight in wheat, which may lead to a potential trade‐off with grain yield (Jones et al., 2018). Therefore, the decipherment and further understanding of genomic regions that affect SL are essential for elucidating the genetic basis of wheat yield and for improving wheat productivity as a whole, which also drives continual improvements in the wheat breeding process.

In recent decades, quantitative trait locus (QTL) mapping related to SL has been extensively studied (Deng et al., 2017; Fan et al., 2019; Jantasuriyarat et al., 2004; Zhai et al., 2016). Primarily, six QTLs related to SL were identified through 110 recombinant inbred lines (RILs) in 2004, accounting for 11%–23% of the phenotypic variation (Jantasuriyarat et al., 2004). Based on a dense genetic linkage map with 10,816 markers, five spike morphological traits were evaluated under nine environments for the 191 RILs and parental lines. A total of 44 QTLs were detected, of which six environmentally stable QTLs for SL were identified on chromosomes 1B, 2B, 2D, 5A, and 7B (Zhai et al., 2016). QTLs for six spike‐related traits were detected under different nitrogen treatments using a high‐density genetic linkage map constructed with polymerase chain reaction (PCR) markers, Diversity Arrays Technology (DArT), and Affymetrix Wheat 660 K single nucleotide polymorphism (SNP) chips; finally, a total of 22 QTLs for SL were detected by inclusive composite interval mapping, which accounted for 2.05%–20.56% of the total SL variation (Fan et al., 2019). A double haploid (DH) population was used for QTL mapping of five spike‐related traits under four different nitrogen supply dates in two locations and years. Seven QTLs were detected in 2017, with each QTL explaining 6.32%–24.2% of the total SL variation (Deng et al., 2017). However, observations have indicated that the results of the QTL mapping showed significant differences among the various materials, genetic backgrounds, and evaluated locations and years (Khahani et al., 2020; W. Wang et al., 2022). Thus, few of the detected QTLs received considerable attention.

Meta‐QTL (MQTL) studies have been used extensively in recent years. MQTL analysis is a relatively novel concept that aims to identify robust, comprehensive, and insightful MQTL regions often linked to trait variations, irrespective of different backgrounds, and narrow down their confidence interval (CI). This process facilitates the identification of candidate genes (CGs) for marker‐assisted selection (MAS) (Goffinet & Gerber, 2000; Soriano & Alvaro, 2019; Veyrieras et al., 2007). An intriguing opportunity to consolidate available QTL information by comparing individual experiments arises from QTL meta‐analysis (Delfino et al., 2019). In addition, it is designed to provide a more comprehensive and statistically robust understanding of a particular phenomenon by comparing and merging data from different sources (Sharma et al., 2023). The involvement of multiple studies can help mitigate bias effects that might be present in independent studies (Salih & Adelson, 2009). In the realm of crop research, the MQTL analysis has been considered a highly effective method, which was vital in identifying consensus QTL regions related to a wide range of economically significant traits. Nowadays, MQTL analysis is applied successfully in many crops for various quantitative traits, such as rice (*Oryza sativa* L.) (Daware et al., 2017; Selamat & Nadarajah, 2021), potato (*Solanum tuberosum* L.) (Danan et al., 2011), maize (*Zea mays* L.) (M. Gupta et al., 2023; W. Wang et al., 2022), cotton (*Gossypium hirsutum* L.) (Said et al., 2015), soybean [*Glycine max* (L.) Merr] (Hwang et al., 2016; Qin et al., 2018; Van & McHale, 2017), and grapevine (*Vitis vinifera* L.) (Delfino et al., 2019). MQTLs have also been identified in wheat for several traits, including tan spot resistance (Y. Liu et al., 2020), resistance to Fusarium head blight diseases (Saini, Chahal et al., 2022; Steiner et al., 2019; Venske et al., 2019), drought tolerance (A. Kumar et al., 2020), nitrogen use efficiency and root system architecture (Saini et al., 2021), quality traits (Gudi et al., 2022; Singh et al., 2022), kernel size (J. Ma et al., 2022), TGW (Griffiths et al., 2009; J. Miao et al., 2022; L. Zhang et al., 2010), and so on. Overall, MQTL analysis will remain a critical tool in the arsenal of modern agriculture, contributing to crop enhancement and food production in a constantly changing world (Sharma et al., 2023).

In the current study, conducting a meta‐analysis for wheat SL based on the results of QTL mapping in previously published studies has enabled the identification of key CGs in the important MQTL interval to pinpoint excellent haplotypes. This study will contribute to providing gene resources for wheat genetic improvement and boost further work on elucidating the intricate genetic framework and cloning of CGs.

**Core Ideas**

- As many as 227 initial quantitative trait loci (QTLs) related to spike length (SL) were collected to conduct the meta‐QTL (MQTL) analysis.
- The 48 MQTLs which harbored 2240 putative candidate genes (CGs) were identified.
- A functional kompetitive allele‐specific PCR (KASP) marker was developed for *TaPP2C‐3B*.
- The haplotype *TaPP2C‐3B‐Hap II* was an elite allelic variation for high SL.

## 2 MATERIALS AND METHODS

### 2.1 Collection of QTLs for SL from independent studies

The exhaustive literature review was conducted to search for the published mapping QTL studies from 2004 to 2022 for QTLs affecting SL in wheat using PubMed (http://www.ncbi.nlm.nih.gov/pubmed), Google Scholar (https://scholar.google.com/), and Web of Science (https://webofscience.clarivate.cn). For each QTL region, collected available QTL information on all essential parameters related to SL of wheat were from the literature, including (1) name of QTL; (2) spike trait information; (3) parents of the mapping population; (4) chromosome location; (5) type of mapping population: F_2_, backcross (BC), DH, RILs, and near‐isogenic lines (NILs); (6) size of mapping population; (7) flanking markers for interval mapping; (8) genetic position of the peak and associated 95% CI; (9) logarithm of odds ratio score (LOD); and (10) the phenotypic variation explained (PVE) or *R* ^2^ values of the QTLs. The LOD score was assumed to be 3 in a few case studies where the LOD values were missing. This decision was based on the previous article, which either reported a *p*‐value statistic or indicated that a minimum LOD score of 3 was considered as the threshold for the QTL analyses (Nagelkerke, 1991). Also, the *R* ^2^ value was regarded as 10 when several QTLs identified in the previous study did not have unambiguous definitions (Saini, Srivastava et al., 2022). When the peak position was missing, the midpoint between the two flanking markers was treated as the position. In addition, for the initial QTLs without flanking markers or CIs, the CIs were then recalculated according to the type and size of the population used in QTL mapping via the following standard formulas: (1) the equation CI = 530/(N × *R* ^2^) was suitable for the F_2_ and BC populations (Darvasi & Soller, 1997), (2) the equation CI = 163/(N × *R* ^2^) was presented by modeling to calculate the 95% CI of RIL and NIL populations (B. Guo et al., 2006), and (3) the equation CI = 287/(N × *R* ^2^) was used for the DH population (Visscher & Goddard, 2004). In the three formulas, CI represents the confidence interval of QTL, while 530, 163, and 287 are the population‐specific constants obtained from various simulations; *N* denotes the size of the QTL mapping population used for QTL analysis; and *R* ^2^ indicates the genetic contribution rate of the initial QTL (Darvasi & Soller, 1997; B. Guo et al., 2006; Venske et al., 2019). In QTL mapping studies, the following three types of main markers were used to generate genetic linkage maps: simple sequence repeat (SSR), DArT, and SNP.

### 2.2 Quantitative trait locus projection and meta‐QTL analysis

According to the criteria mentioned above, the collected information, including the initial QTL name, CI, peak position, chromosome distribution, LOD score, PVE, and the intensive enough reference map, was compiled from published studies as input files to create a consensus map and conduct further MQTL analysis. It is worth noting that QTLs without being projected onto the consensus map were discarded as they lacked common markers on the consensus map or were located outside the consensus map. Based on the integrated consensus map and projections of initial QTLs obtained from different genetic or environmental backgrounds, MQTL analysis was conducted for each chromosome individually using the software BioMercator v4.2.3. Two different methods were employed depending on the number of initial QTLs (Sosnowski et al., 2012). The meta‐QTL analysis first ascertains the number of initial QTLs on each chromosome from various populations. The following are detailed descriptions of both methods: For the number of initial QTLs less than 10 on each chromosome, the best model with the minimum Akaike information criterion (AIC) value was selected from five models with different AIC values (1, 2, 3, 4, or *N*) to identify MQTL positions of consensus (Goffinet & Gerber, 2000). The method proposed by Veyrieras et al. (2007) was employed when the number of initial QTLs on a chromosome was at least 10. Based on this approach, meta‐analyses were conducted for individual chromosomes using a two‐step method available in the software. In the first step, the collected QTLs on individual chromosomes were clustered using default parameters. The number of potential MQTLs per chromosome was then estimated based on the following five selection criteria: AIC, corrected Akaike information criterion, Akaike information criterion 3, Bayesian information criterion, and approximate weight of evidence. Among the five models, the model with the lowest values of the selection criteria in at least three models was chosen as the optimal MQTL model for the subsequent stage of meta‐analysis. In the second step, the 95% CI and the positions of each MQTL were determined according to the optimal model selected in the previous step. The genomic region with more than two initial QTLs was defined as a MQTL. The LOD score and PVE values of each MQTL were calculated as the average of the LOD and PVE values of the initial QTLs involved.

### 2.3 Identification of putative CGs within MQTL regions

The putative CGs were identified as the genes located within the MQTL intervals, which could be determined by the physical positions of the flanking markers of the MQTL. The next step was to manually search for the flanking markers within the target MQTLs on the final consensus map based on the 95% CI and the positions of each MQTL. Based on the positions of flanking markers, the physical positions of each MQTL could be determined on the Chinese Spring wheat reference genome using the Triticeae Multi‐omics Center (http://wheatomics.sdau.edu.cn) (S. Ma et al., 2021) annotated by IWGSC_v1.1_HC_gene. It was possible that the physical positions of the flanking markers could not be found. The GrainGenes database (https://wheat.pw.usda.gov/GG3) (Blake et al., 2019) or the DArT database (https://www.diversityarrays.com) were used to obtain their sequences. The available sequence information was aligned to the wheat reference genome at the Triticeae Multi‐omics Center, followed by the steps to determine the physical location of the flanking markers using the BLASTN program.

The MQTLs are considered as potential genomic regions likely to harbor putative CGs for the target traits (Pal et al., 2021). According to the annotation of the wheat reference genome, CGs could be further screened. In the present study, three methods were performed to identify the CGs within MQTL regions. (1) The strategy of wheat‐rice orthologous comparison was used to identify the putative CGs in the MQTL regions. In addition, the homologous genes in wheat were acquired from the Triticease‐Gene Tribe (http://wheat.cau.edu.cn/TGT/) (Y. Chen et al., 2020) based on the IWGSC RefSeq v1.1. (2) The peak physical position of the MQTLs was calculated according to the method proposed by Saini (Saini, Srivastava et al., 2022). (3) The MQTLs with at least two overlapping initial QTLs, a physical distance of <20.0 Mb and a genetic distance of <1.0 cM, were referred to as core MQTLs. The physical positions were then entered into the search toolbox of the “Gene” in the WheatGmap database (http://www.wheatgmap.org/) (L. Zhang, Dong et al., 2021) to obtain details of gene models (locus ID information and functional descriptions) corresponding to MQTL regions.

### 2.4 Transcriptomic data integration of CGs within MQTL regions

Analyzing the expression characteristics of CGs in various tissues and developmental stages is crucial for determining their function. The expression visualization and integration platform (http://www.wheat‐expression.com) (Borrill et al., 2016), which includes expression data from up to 18 tissues containing the wheat growth period, was utilized in the current study. This expression database included the following details: (1) grain tissue‐specific developmental time course (Pearce et al., 2015; Pfeifer et al., 2014), (2) developmental time‐course of Chinese Spring (Choulet et al., 2014), (3) Chinese Spring seedling (leaves and roots) and spikes at anthesis (Maqbool et al., 2023), and (4) leaves and roots of Chinese Spring wheat at the seedling stage (Clavijo et al., 2017) and gene expression during flag leaf senescence (Borrill et al., 2019). The gene ontology (GO) and Kyoto Encyclopedia of Genes and Genomes (KEGG) analyses were conducted using the GENEDENOVO cloud platform (https://www.omicshare.com). For transcriptional expression analysis, only the CGs showing at least 2 transcripts per million (TPM) expressions were considered in this study (Wagner et al., 2013). The expression characteristics of CGs were displayed through a heat map of TPM using the TBtools software.

### 2.5 Polymorphism identification and development of kompetitive allele‐specific PCR marker

The sequence alignment for the key CGs was performed to identify the SNPs in the coding regions and promoter using the Wheat Union website (http://wheat.cau.edu.cn/WheatUnion/) (W. Wang et al., 2020). The variant information query module was used to download the genotype, with both the upstream and downstream extensions set to 2000 bp. The website PlantPAN 3.0 (http://PlantPAN.itps.ncku.edu.tw/) (Chow et al., 2019) was used to predict the quantity and variety of cis‐regulatory elements in the promoter. The association analysis of the key CGs was conducted using the natural population sown at Zhuanglang farm station, Gansu, China (105°98′ E, 35°37′ N, altitude 2110 m) in 2021–2022 (E1) and 2022–2023 (E2); at Tongwei farm station, Gansu, China (105°19′ E, 35°11′ N, altitude 1750 m) in 2021–2022 (E3); and at Tianshui farm station, Gansu, China (105°69′ E, 34°6′ N, altitude 1550 m) in 2022–2023 (E4). Each wheat line was sown in late September and harvested in early July of the following year. A randomized complete block design was conducted with three replications, where the row length of each plot was 1 m and the row spacing was 20 cm, and 30 seeds were sown per row.

## 3 RESULTS

### 3.1 Main features for SL QTL studies and projection of initial QTLs

**FIGURE 1:**
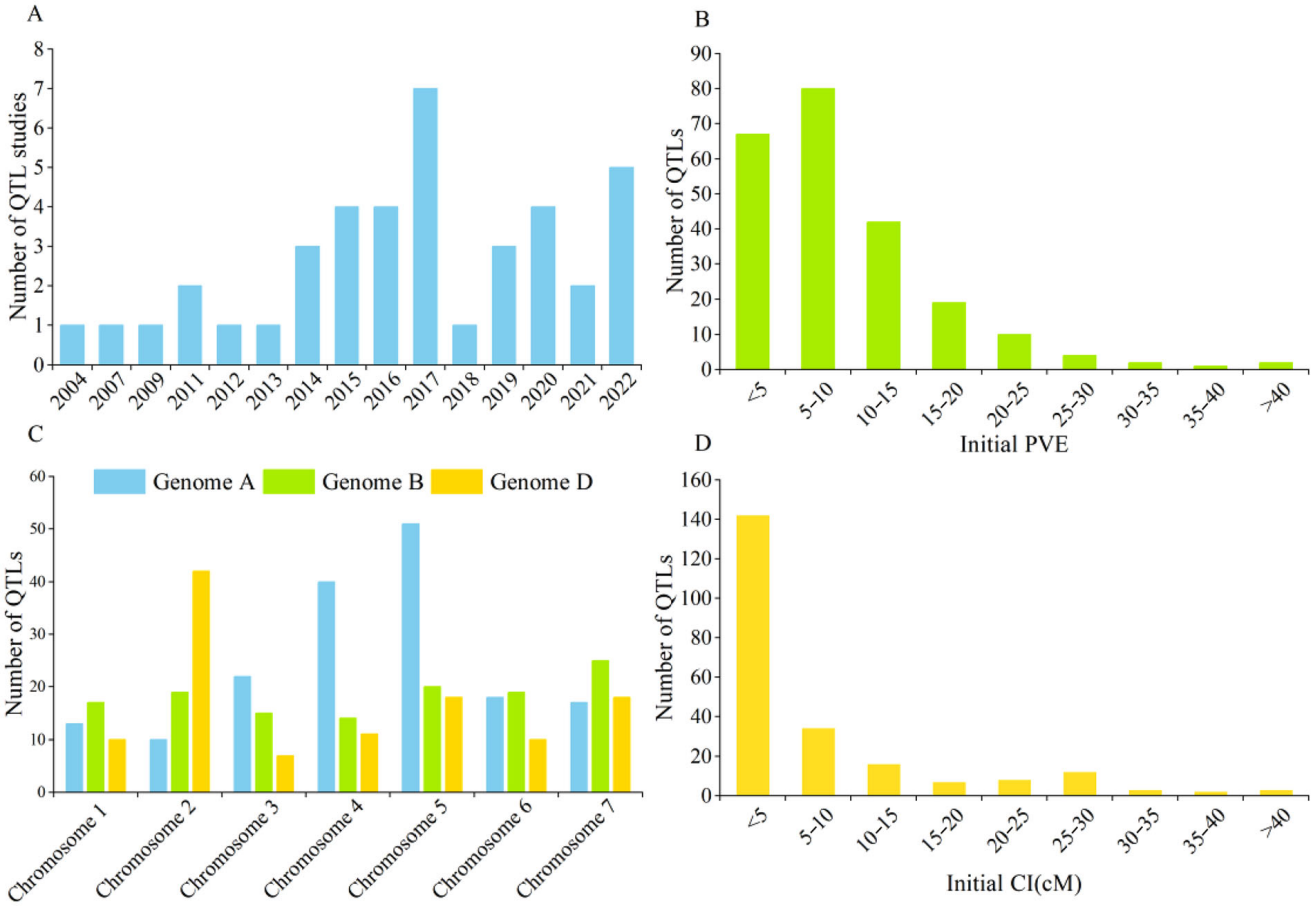
Characteristic features of quantitative trait loci (QTLs) for spike length (SL) in published QTL mapping studies. (A) The temporal distribution of previous QTL studies for SL. (B) Frequencies of QTLs with individual levels of phenotypic variation explained (PVE). (C) The number of QTLs in wheat sub‐genomes. (D) Confidence intervals (CIs) of the initial QTLs.

Between the years 2004 and 2022, all published QTL studies for SL were retrieved from the literature using appropriate keywords, leading to the selection of 41 independent studies (Figure 1A). These QTL studies involved 46 different cross populations, with an average of 168 offspring per study, ranging in size from 82 to 310. The majority of these studies were published between 2014 and 2022, with only a few published before 2014. Among the 46 mapping populations used in 41 different studies over the past decades, more than 80.43% were RIL populations. These lines of the permanent mapping populations were genetically stable enough to be used for phenotyping yield and yield‐related traits for years under different environmental conditions (Table S1). Here, a total of 416 initial QTLs were available in the reported 40 studies. These 416 reported QTLs were distributed unevenly among all 21 wheat chromosomes, with an average of 20 QTLs per chromosome. Chromosome 5A harbored the highest number of QTLs with 51, followed by chromosome 2D with 42 and 4A with 40, chromosome 3D had the lowest number with only seven (Figure 1C). As for the PVE by a single QTL, it varied from 0.54% to 54.20% (with a mean of 8.98%), with most of the QTLs explaining PVE ≤ 20% (Figure 1B). In addition, the 95% CI of initial QTLs for SL ranged from 0.01 to 80.41 cM with an average of 8.20 cM. Approximately 313 (75.24%) of the collected initial QTLs had a CI of less than 10 cM, and 362 (87.02%) had a CI of less than 20 cM (Figure 1D).

**FIGURE 2:**
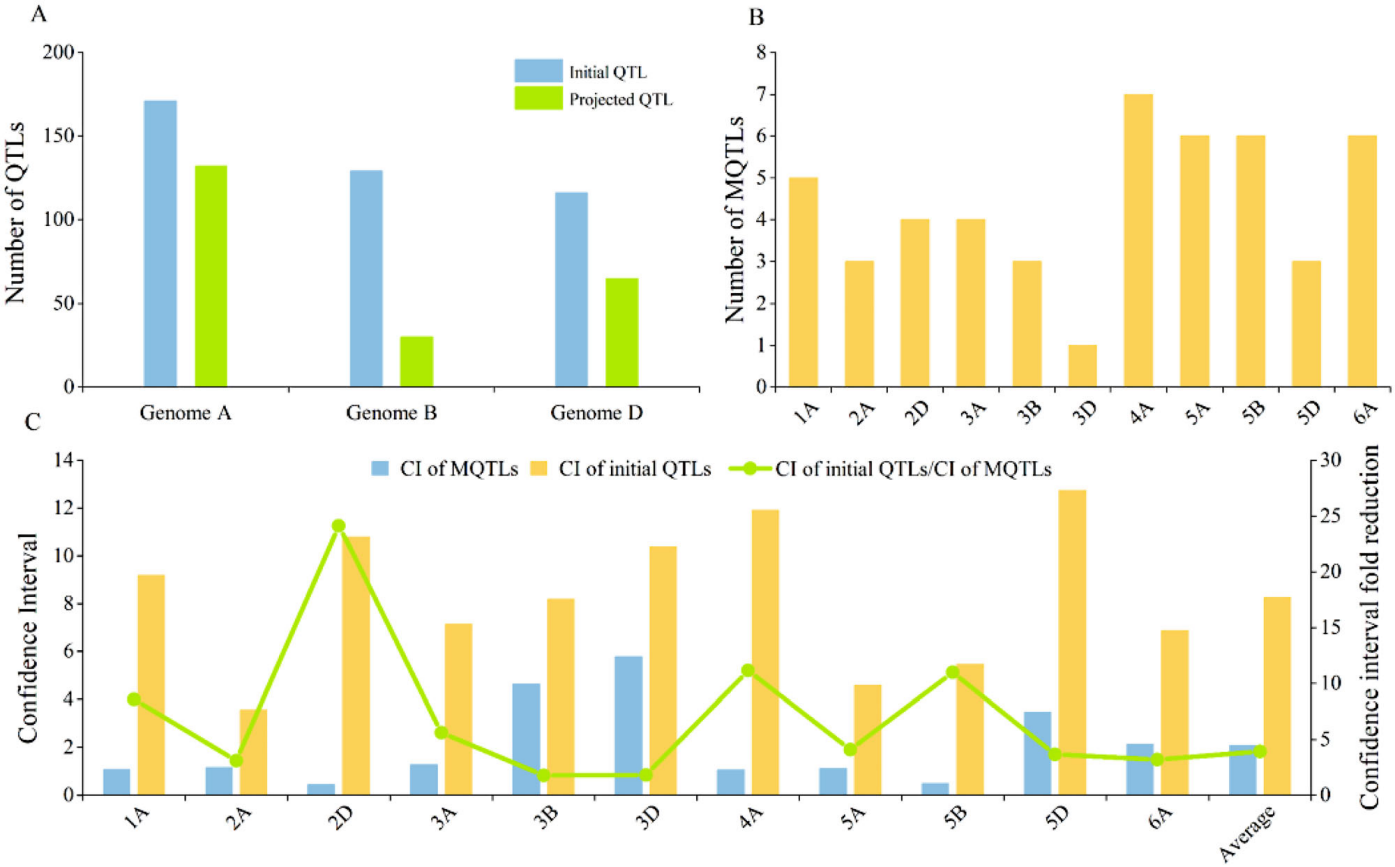
Summary information of meta‐QTL (MQTLs) obtained in MQTL analysis. (A) The difference between initial and projected quantitative trait loci (QTLs). (B) The distribution of identified MQTLs on wheat chromosomes. (C) The reduction degree of QTL 95% confidence interval (CI) after meta‐QTL analysis

Out of the 416 initial QTLs, only 227 (54.57%) were available for further MQTL analysis using Biomercator V4.2.3 because the remaining 189 lack common flanking markers with the markers in the consensus map. This map comprised 14,548 markers, including SSR, DArT, SNP, and other types of markers, with a total length of 4813.72 cM, ranging from 155.6 to 350.11 cM in the 21 linkage groups. Consequently, chromosome 5A had the highest number of projected QTLs (44), while chromosomes 4A and 2D had the lowest number (36 and 28) with an average of 13. Also, these QTLs were projected on the three sub‐genomes, with 132 (58.15%) located on sub‐genome A, only 30 (13.22%) on sub‐genome B, and 65 (28.63%) on sub‐genome D, respectively (Figure 2A).

### 3.2 Detected MQTL related to SL and their distribution on wheat chromosomes

**FIGURE 3:**
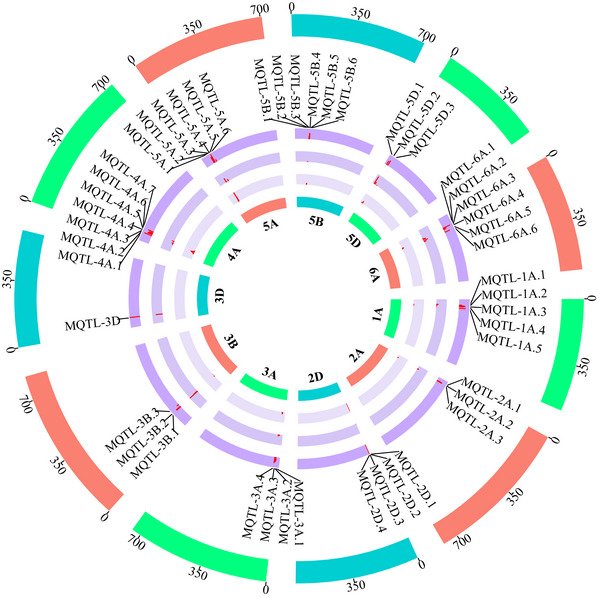
The chromosome distribution of 48 meta‐QTLs (MQTLs) for spike length (SL) via MQTL analysis. The circles from outside to inside represent the physical chromosome distance (Mb), the position of 48 MQTLs, the phenotypic variation explained (PVE), confidence interval (CI), and the number of initial QTLs, respectively.

MQTLs with more than two initial QTLs detected in multiple environments were considered to be more confident and stable, regardless of genetic background and environment. In this study, 48 MQTLs were identified through the MQTL analysis using 416 initial QTLs, representing an 88.46% reduction in the number of initial QTLs (Figures 2B and 3, Table S2). These MQTLs were located on 11 different wheat chromosomes, including chromosomes 1A, 2A, 2D, 3A, 3B, 3D, 4A, 5A, 5B, 5D, and 6A, with an average of four MQTLs per chromosome (Figure 2B). Chromosome 4A harbored the largest number of MQTLs with six MQTLs, while chromosome 3D had the lowest number of MQTLs with one MQTL. Average MQTLs for three sub‐genomes also differed, with the lowest number of MQTLs found in the D sub‐genome (eight MQTLs), followed by the B sub‐genome (nine MQTLs), and the A sub‐genome (31 MQTLs). Among the 48 identified MQTLs, all of them comprised at least two initial QTLs, with 83.3% (40/48) of MQTLs including three or more initial QTLs and 18.75% of MQTLs being made up of 10–26 initial QTLs. Among them, four MQTLs were identified from the more than 20 initial QTLs, including MQTL‐2D.4 (20), MQTL‐2D.3 (21), MQTL‐5A.3 (22), and MQTL‐5A.4 (26). In light of these MQTLs that contained multiple initial QTLs which were not being detected in only one environment, MQTLs were more reliable and stable.

The MQTL analysis resulted in a reduction in the 95% CI of MQTL compared to the average CI for initial QTL located in MQTL regions (Figure 2C). Among different chromosomes, there was a significant difference in the mean of 95% CI. The 95% CI of the identified MQTLs, ranging from 0.01 to 9.38 cM with an average of 1.59 cM, was a 5.16‐fold reduction than that of the initial QTLs with 8.20 cM. The mean CI for MQTLs on chromosome 2D was reduced by 95.86% compared to initial QTLs, but only reduced by 43.54% on chromosome 3B. About 56.25% (27/48) of MQTLs had CI less than 1 cM, and the physical position varied from 1.99 Mb for MQTL‐3D to 150.51 Mb for MQTL‐3B.2 with an average of 42.26 Mb per MQTL. To further refine the MQTL, the 11 MQTLs were identified as core MQTLs. Moreover, the physical distances of the core MQTLs ranged from 3.21 Mb for MQTL‐1A.5 to 18.87 Mb for MQTL‐5A.2, while the genetic distance ranged from 0.20 cM for MQTL‐5A.2 to 0.99 cM for MQTL‐4A.2. These MQTL regions above could be utilized to further discover key CGs for SL.

### 3.3 Identified CGs for SL underlying MQTL regions

The advantage of MQTL analysis is that it narrows the CI, which consequently results in increasing the accuracy of putative CGs mining. With the aim of better understanding the genetic mechanism of SL, the decipherment o CGs was conducted based on the physical distance of the identified MQTLs. To further explore the CGs for controlling SL in wheat, an extensive search on the reported genes affecting SL in rice was performed, accounting for 58 functional genes. Consequently, a total of 2409 putative CGs were identified within MQTL regions, which comprised 2240 unique CGs after eliminating duplicate CGs in overlapping MQTLs (Table S3). The highest number of 254 CGs were identified in MQTL‐5A.2, followed by 204 in MQTL‐3A.2, while the lowest number of only four genes were detected in MQTL‐5A.3. Furthermore, more than 100 CGs were identified in eight MQTL regions, including MQTL‐3A.2, MQTL‐5A.2, MQTL‐5A.4, MQTL‐5A.5, MQTL‐5B.2, MQTL‐5B.3, MQTL‐5B.4, and MQTL‐6A.1. Less than 10 CGs were present in five MQTL regions, such as MQTL‐1A.1, MQTL‐2A.2, MQTL‐5A.3, MQTL‐5A.6, and MQTL‐5B.1. Among these identified MQTLs, such as MQTL‐2D.1, MQTL‐2D.2, MQTL‐2D.3, MQTL‐2D.4, MQTL‐4A.3, MQTL‐4A.4, MQTL‐5A.3, MQTL‐5A.4, and MQTL‐5A.5, which have a large number of initial QTLs for SL, they possessed 12, 28, 73, 24, 41, 20, 4136, and 197 putative CGs, respectively.

Multiple putative CGs related to SL determined in previous individual studies in wheat were also found to be located in the different MQTL regions selected in the present study, such as SQUAMOSA promoter‐binding protein‐like, MADS‐box transcription factor, F‐box protein, E3 ubiquitin‐protein, U‐box domain‐containing protein, and so on. In addition, a large number of the orthologous wheat genes of rice genes were also identified in the MQTL regions which are involved in various functions, including the basic leucine zipper (bZIP) transcription factor, short‐grain‐like protein 1, apetala2/ethylene‐responsive element binding factor, DUF1645 domain‐containing protein, auxin/indole‐3‐acetic acid protein, glycosyl transferase, PLUS3 domain‐containing protein, and so forth. The above results demonstrated the significance of the MQTL identified in this study, which warranted further discussion in the following section.

### 3.4 In silico pattern for putative CGs located in the MQTL regions

**FIGURE 4:**
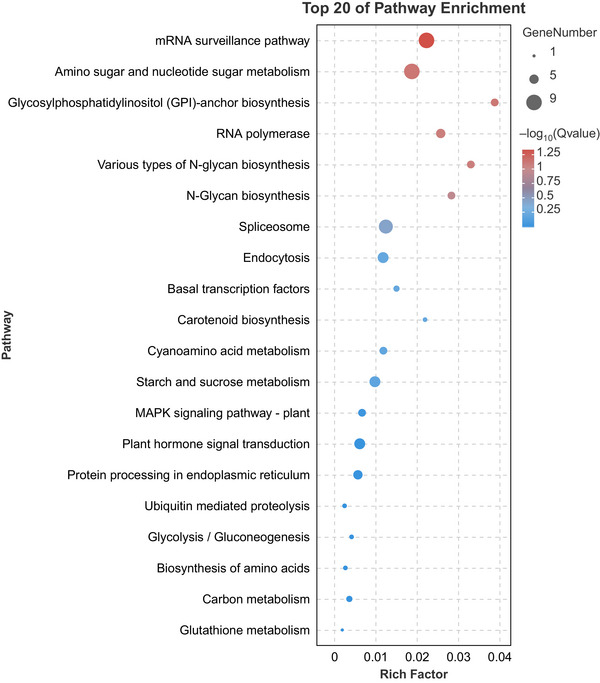
Top 20 Kyoto Encyclopedia of Genes and Genomes (KEGG) enrichment pathways for 567 putative candidate genes (CGs) from meta‐quantitative trait locus (MQTL) regions.

**FIGURE 5:**
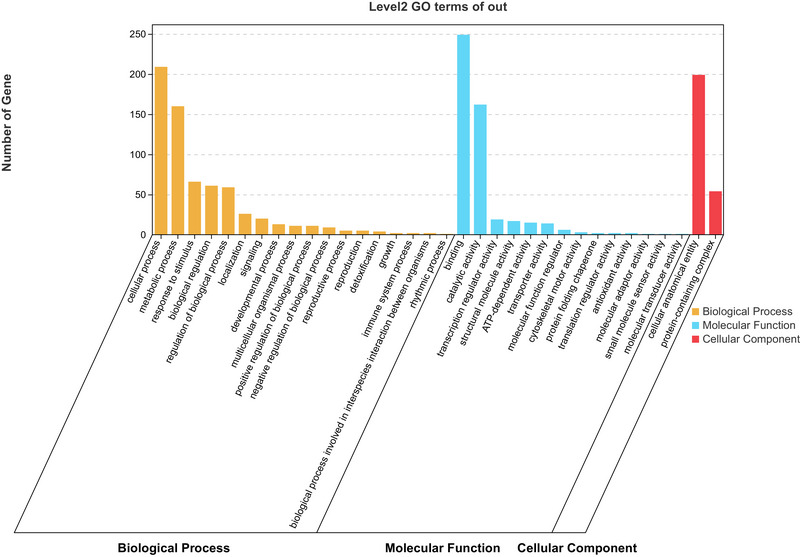
Level 2 gene ontology (GO) terms for 567 putative candidate genes (CGs) from meta‐quantitative trait locus (MQTL) regions.

As for the 2240 putative CGs obtained, the top priority is to perform expression analysis. Consequently, 567 CGs with >2 TPM, mainly expressed in the spike, were considered as genes for further analysis. As a further approach to reduce the number of putative CGs, the CGs obtained from the MQTL regions were integrated with molecular data obtained from the previous user‐friendly in silico transcriptomic platform via GO enrichment and KEGG pathway analyses were conducted. Many putative CGs with similar available information were identified, including 136 for protein with F‐box family, 44 for protein kinase, 40 for protein with cytochrome P450 family, 35 for transmembrane protein, 31 for zinc finger protein, 28 for serine/threonine‐protein kinase, and so on. Among the identified MQTL regions, specific gene superfamilies were also found, such as the RING/U‐box superfamily protein, bHLH DNA‐binding superfamily protein, ARM repeat superfamily protein, and so forth. KEGG enrichment analysis showed that these putative CGs were significantly enriched in mRNA surveillance, amino sugar and nucleotide sugar metabolism, glycosylphosphatidylinositol‐anchor biosynthesis, and RNA polymerase pathways, with the highest number of putative CGs involved in the mRNA surveillance pathway (Figure 4). In addition, GO analysis revealed a variety of GO terms, with some of the most indispensable and abundant GO terms being those related to all three categories (Figure 5). Among biological processes, the CGs were most enriched in metabolic processes (209 CGs) and cellular processes (160 CGs). As for molecular functions, the CGs were most enriched in binding (249 CGs) and catalytic activities (162 CGs). In terms of cellular components, the CGs were significantly involved in the cellular anatomical entity (199 CGs) and protein‐containing complex (54 CGs).

**FIGURE 6:**
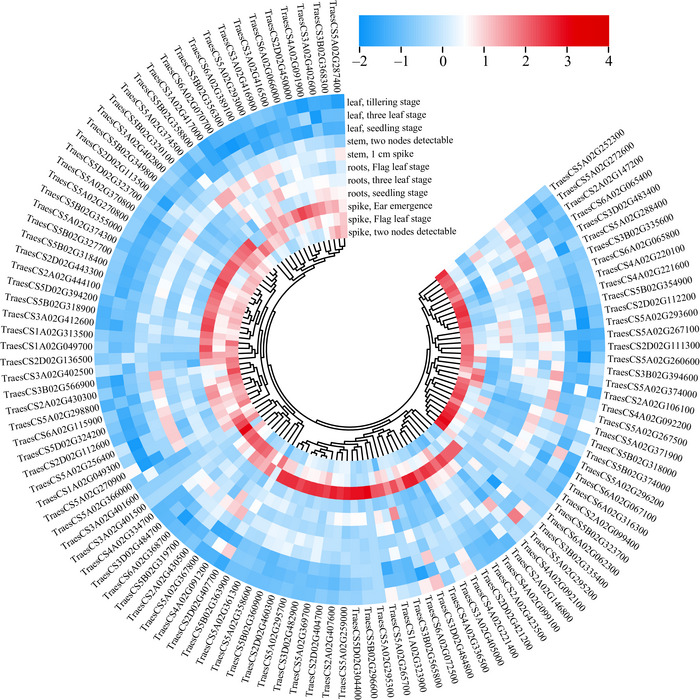
Expression patterns of 114 putative candidate genes (CGs) in four different tissues. Expression values were normalized by log_2_ (transcripts per million [TPM] + 1).

The 114 putative critical CGs within MQTL regions affecting SL, identified through enrichment analysis of GO and KEGG pathways, were selected for further assessment. These genes were analyzed for expression at various stages of wheat growth and development. Expression analysis of 114 putative critical CGs allowed the identification of 95 CGs with >5 TPM expressions, including 70 CGs with >10 TPM and 39 CGs with >20 TPM expressions in spike. Hierarchical clustering of all these 114 putative critical CGs led to the identification of four clusters containing differentially expressed genes. Cluster I contained 28 CGs which were highly expressed in the spike at the two nodes detectable stage. Cluster II, consisting of 29 CGs, showed high expression levels in the spike during the ear emergence stage. Cluster III possessed the remaining 57 CGs, which were mostly expressed among all three stages, such as spikes at the ear emergence stage, flag leaf stage, and two nodes detectable stage, showed the slight differential expression (Figure 6). The gene *TraesCS3B02G394600* (*TaPP2C*‐*3B*) was highly expressed at the spike two nodes detectable stage, while its homologous gene in rice (*OsPP2C09*) showed a positive effect on agronomic traits.

### 3.5 Dominant haplotype analysis and molecular marker development

**FIGURE 7:**
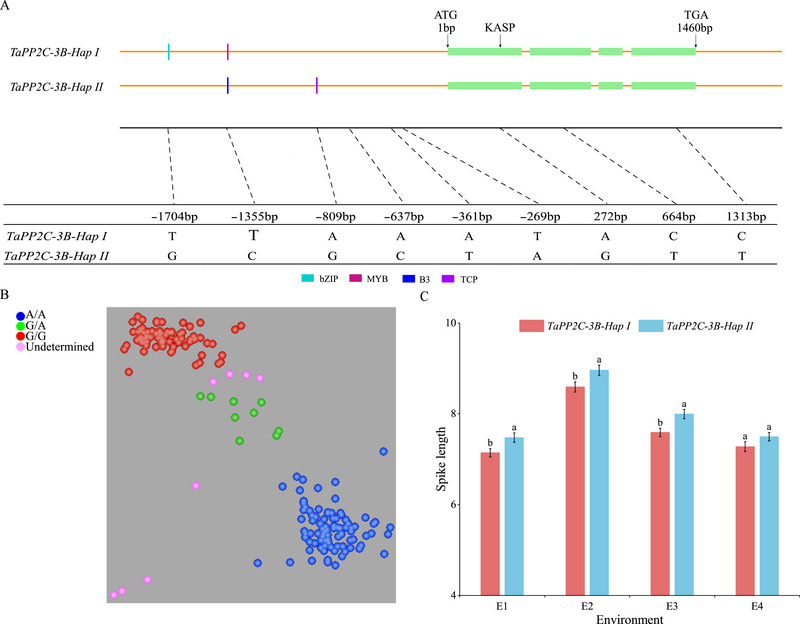
(A) Schematic *TaPP2C*‐*3B* gene structure and main cis‐element distribution of promoter. (B) kompetitive allele‐specific PCR (KASP) genotyping scatter cluster diagram of *TaPP2C*‐*3B*. (C) Association analysis of *TaPP2C*‐*3B* allelic variation and spike length in different environments. bZIP, basic leucine zipper; MYB, v‐myb avian myeloblastosis viral oncogene homolog; B3, ABSCISIC ACID INSENSITIVE 3; TCP, teosinte branched1/Cycloidea/Proliferating cell factor.

Based on the variations revealed by genomic sequence data of 681 wheat varieties from the Wheat Union website (http://wheat.cau.edu.cn/WheatUnion/) (W. Wang et al., 2020), the polymorphic SNP loci on the key CGs *TaPP2C*‐*3B* were identified. Multiple sequence alignment revealed three SNPs were identified in the coding region located at 272 (A/G), 664 (C/T), and 1313 (C/T) bp, while six SNPs were detected in the promoter sequence of *TaPP2C*‐*3B* which were located at −269 (T/A), −361 (A/T), −637 (A/C), −809 (A/G), −1355 (T/C), and −1704 (T/G) bp. On account of these variable sites, two haplotypes of *TaPP2C*‐*3B* were formed and named *TaPP2C*‐*3B*‐*Hap I* and *TaPP2C*‐*3B*‐*Hap II* (Figure 7A). A kompetitive allele‐specific PCR (KASP) marker was designed to distinguish between the *TaPP2C*‐*3B*‐*Hap I* and *TaPP2C*‐*3B*‐*Hap II* haplotypes based on an SNP at 272 bp (A/G) to differentiate the wheat germplasms from two haplotypes (Figure 7, Table S4). The 293 accessions were genotyped using the KASP marker (Figure 7B). Bioinformatic analysis revealed that three SNPs located at the promoter of *TaPP2C*‐*3B* participate in the formation of several transcription factor binding sites, including bZIP, MYB, B3, and TCP (Figure 7A). Therefore, the unique transcription factor binding site contained in the promoter sequence of *TaPP2C*‐*3B*‐*Hap II* may account for its superiority. The association analysis was conducted to detect the impact of *TaPP2C*‐*3B* allele variation on agronomic traits in wheat. The results revealed that *TaPP2C*‐*3B* was significantly associated with the SL in three environments, while the SL of *TaPP2C*‐*3B*‐*Hap II* was consistently higher than that of *TaPP2C*‐*3B*‐*Hap I* (Figure 7C). However, there was no significant association between *TaPP2C*‐*3B* and GNS, spikelet number per spike, and TGW observed in any environments. Thus, the haplotype *TaPP2C*‐*3B*‐*Hap II* could be a potential haplotype for improving SL.

### 3.6 Selection of *TaPP2C*‐*3B* haplotypes during wheat breeding in China

**FIGURE 8:**
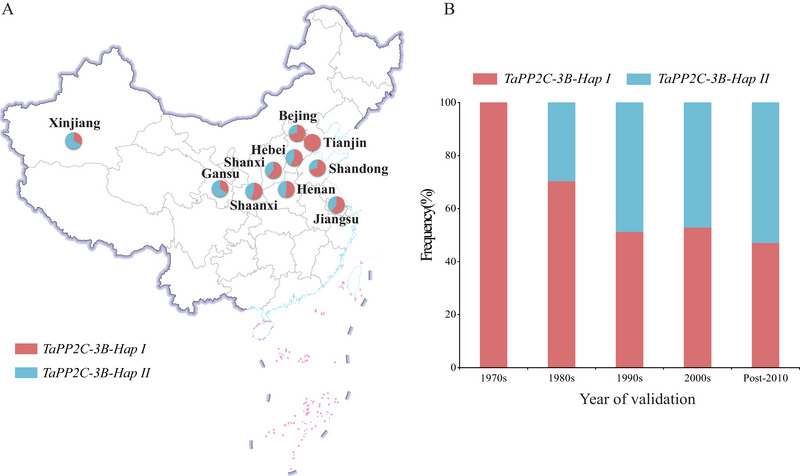
Selection of *TaPP2C*‐*3B* haplotypes in wheat breeding in China. (A) Geographic distribution of varieties with *TaPP2C*‐*3B* haplotypes in China. (B) Variation of the proportion of haplotypes of *TaPP2C*‐*3B* with breeding age.

The course of wheat breeding supported the gradual accumulation of superior haplotypes. The geographic distribution of the two alleles of the *TaPP2C*‐*3B* was evaluated using 272 wheat accessions (Table S5). It is suggested that the selection pressure on haplotypes in the different provinces was not as strong as expected. The frequencies of the dominant haplotype *TaPP2C*‐*3B*‐*Hap II* with high SL were generally low, except in two provinces: Gansu (65.00%) and Xinjiang (66.67%) (Figure 8A). To further assess whether the *TaPP2C*‐*3B*‐*Hap II* was positively selected during the process of wheat breeding in China, the frequencies of *TaPP2C*‐*3B* haplotypes in the historical population of 192 accessions were detected at 10‐year intervals (Table S6). The frequency of *TaPP2C*‐*3B*‐*Hap II* increased from the 1970s (0%) to the 1990s (48.78%), then maintained a relatively stable average level of about 49.62% from the 1960s to the 1990s (Figure 8B). It was suggested that favored haplotypes *TaPP2C‐3B‐Hap II*, with considerable potential for further utilization, have undergone positive selection during the wheat breeding process.

## 4 DISCUSSION

### 4.1 Characteristics of QTL and MQTL regions related to wheat SL

The availability of independent QTL mapping studies for yield and other yield‐related traits has provided a solid foundation for breeding high‐yielding wheat varieties through MAS (Sharma et al., 2023; Yang et al., 2021). In the past decades, a wide range of QTL mapping studies have been conducted due to its relative simplicity and compelling concept, aiming to reveal the genetic determinism associated with traits in wheat (J. Hu et al., 2020; H. Liu et al., 2022; Sharma et al., 2023; Wei et al., 2022; X. Xu et al., 2022). In this study, 416 initial QTLs related to SL were projected onto the 21 chromosomes of wheat from 40 individual QTL mapping studies. In agreement with previous studies on grain yield and other related traits, these initial QTLs were unevenly distributed on chromosomes, with the sub‐genome A and sub‐genome B harboring the vast majority of them (A. Kumar et al., 2020; Saini, Srivastava et al., 2022; Yang et al., 2021). However, only 227 available QTLs were used for conducting the meta‐analysis. While the sub‐genome B contained only 13.22% (30/227) of the QTLs, this observed phenomenon could be attributed to the deficient common markers on the sub‐genome B.

The large number of QTLs could be detected, but it was by no means certain when working in breeding programs due to the high susceptibility of its results to the type of mapping populations, environment, reference map, and statistical models used (Gudi et al., 2022; Khahani et al., 2020). Recent trends have indicated that meta‐analysis could be held accountable for the identification of robust and consistent MQTL regions associated with trait variations in various QTL mapping studies and reduced the CI, which would therefore seem to facilitate the identification of CGs (Delfino et al., 2019; Soriano & Alvaro, 2019). Previously, a meta‐analysis for wheat SL identified 33 MQTLs based on 87 initial QTLs. Among them, 24 MQTLs contained less than two initial QTLs and 13 MQTLs were composed of only one initial QTL (Yang et al., 2021). However, the number of initial QTLs for meta‐analysis was proportional to the precision of the results of the meta‐analysis (Quraishi et al., 2017; Yang et al., 2021). In the present study, 48 MQTLs were identified through a meta‐analysis based on the modified AIC value (Goffinet & Gerber, 2000; Veyrieras et al., 2007). Among these MQTLs, 52.08% (25/48) MQTLs were composed of more than five initial QTLs, with 18.75% (9/48) MQTLs containing more than 10 initial QTLs which was larger than in earlier meta‐analysis (Amo & Soriano, 2022; M. Gupta et al., 2023). In addition, the conformance of the previous MQTL analysis for SL and other yield‐related traits with this study was contrasted in detail based on the physical position. For example, the 13 MQTLs contributing to SL confirmed in this study were colocated with 11 MQTLs identified in the previous study on SL (Yang et al., 2021). In addition, the MQTL‐2A.1 was colocated with MQTL2A.3 affecting spike‐related traits, grain weight, grain number, and grain morphology‐related traits (Saini, Srivastava et al., 2022), as well as colocated with MQTL‐2A‐1 controlling grain weight, grain filling rate, grain yield, grain number, and days to maturity (Yang et al., 2021). Similarly, the MQTL‐5A.1 was colocated with MQTL‐5A‐2 for TN, grain yield, and flowering date, while MQTL5A.4 affected days to heading, grain morphology‐related traits, and plant height (Saini, Srivastava et al., 2022; Yang et al., 2021). The MQTL analysis for TGW was performed, and finally, 394 initial QTLs were refined into 67 MQTLs. Among them, 27 MQTLs were found to colocalize with the MQTL for SL identified in the current study (J. Miao et al., 2022). Given that MQTL for SL showed strong correlations with yield‐related traits, these MQTLs with a higher level of confidence may be considered as pleiotropic regions that could improve breeding efficiency for multiple traits effectively.

### 4.2 Putative CGs available for SL within MQTL regions

Various prominent CGs affecting wheat SL have been accurately confirmed in MQTL regions. The wheat *TaSPL14* (*TraesCS5A02G265900*) was identified in MQTL‐5A.2, and its knock‐out plants exhibited decreased plant height, SL, spikelet number, and TGW, which was similar to *OsSPL14* knock‐outs in rice. To understand how *TaSPL14* regulates spike development, an RNA‐seq experiment was conducted on young spikes (20–30 mm) performed from wild‐type Fielder and *TaSPL14* knock‐out line implied that *TaSPL14* regulated spike development through genes associated with ethylene response (J. Cao et al., 2021). Among the 47 CGs identified, 23 were located in the MQTLs regions identified in the present study and predicted for SL through integration of QTL mapping and WGCNA. Notably, *TaSINA*‐*like*, *TaHistone H3.2*, and *TaU*‐*box*‐*like* exhibited a higher degree of network, suggesting their potential significant regulatory roles in SL (Wei et al., 2022). Based on expression patterns and orthologous gene functions, four genes within the physical interval were regarded as potential CGs involved in the spike development which were located in MQTL‐2D.1 (M. Wang et al., 2023). Among them, *TraesCS2D02G060000* exhibited high and specific expression in spike, encoding a Sec1/munc18‐like protein, a key regulatory in membrane fusion and associated with vesicle fusion mechanisms during cell division (Rathore et al., 2019); *TraesCS2D02G060100* encoded a cleavage and polyadenylation specificity factor subunit, known to be involved in floral organ morphogenesis in *Arabidopsis thaliana* (Xie et al., 2015); *TraesCS2D02G060400* encoded F‐box domain protein related to floral transition, spike and seed development, and photomorphogenesis and crop yield (Gao et al., 2022; Jain et al., 2007; H. Li, Wei et al., 2020); and *TraesCS2D02G060600* encoded a WD40 repeat protein involved in flowering and spike development heading date in rice (Jiang et al., 2018), grain number, and grain yield in maize and rice (W. Chen, Chen et al., 2022), and seed development in wheat (R. Hu et al., 2018). MQTL‐6A.2 also identified a gene *TraesCS6A02G090300* that was predicted as CGs for SL (T. Li et al., 2021) encoding a eukaryotic translation initiation factor 3 subunit F and its orthologous gene in rice(*OseIF3f*), which played significant role in rice development and reproductive processes, and affected plant height, SL, internode length, and seed size in rice (W. Wang et al., 2016). The conduction of genome‐wide association study for wheat spike‐related traits predicted CGs responsible for SL, kernels per spike, and spikelet number (J. Liu et al., 2018), of which 38, 50, and four CGs for SL were located in MQTL‐2A.2, MQTL‐6A.2, and MQTL‐2D.4, respectively. Thus, finding MQTLs that corresponded to CGs predicted in previous studies was crucial for the discovery of novel CGs that could be used for improving spike characteristics in wheat.

Several studies on the functional analysis of genes affecting SL in wheat have also been conducted in the past few years. A recent study revealed that a SL regulatory QTL located in an interval (159.4–167.2 Mb) on chromosome 7A, named *TaSL1*, also participated in the regulation of kernel length (Wei et al., 2022). The identification of a highly expressed RING finger E3 ubiquitin ligase gene, *TaAIRP2*‐*1B* (*ABA‐insensitive RING protein 2*), in wheat spike showed that *TaAIRP2*‐*1B* was significantly related to SL under different conditions, coupled with haplotype *Hap‐1B‐1* of *TaAIRP2*‐*1B* possessing a longer spike than that of *Hap‐1B‐2* (J. Zhang et al., 2023). *TraesCS2A01G547800* was annotated to encode an auxin response factor, consistent with its close homology to the *ARF12* gene in rice which was related to plant height development (Y. Li, Li et al., 2020), and hence designated as *TaARF12* (A. Li, Hao et al., 2022). The transgenic lines of *TaARF12*‐*KO* by RNAi were further reduced by up to 20.7%, which occurred at each internode, while spikes of *TaARF12*‐*KO* plants became longer resulting in a significant increase in seed per spike (A. Li, Hao et al., 2022). The correlation analysis demonstrated that *TaIAA15*‐*3B* was related to plant height, SL, and TGW in wheat, and *Hap*‐*II* of *TaIAA15*‐*3B* was identified as a superior haplotype and was positively selected in a breeding program in China (F. Li, Yan et al., 2022). The *TaCCA1*‐*OE* transgenic wheat lines appeared to disrupt circadian rhythmicity, decreased chlorophyll and starch content, and reduced biomass at the seedling stage, also reduced SL, GNS, and grain size at the maturity stage (Gong et al., 2022). When the gene *TaSPL13*‐*2B* was transferred into rice, it improved the panicle architecture in transgenic rice, showing remarkable increases in SL, the number and length of primary branches, and the number of secondary branches (L. Li, Shi et al., 2020). The above genes were confirmed to regulate the process of wheat spike development and finally affected SL. However, it was unfortunate that these genes were not located in the MQTL regions identified in this study. A following reasonable explanation could be responsible for this phenomenon that there is a need to supplement novel adequate initial QTLs for SL in wheat on all 21 chromosomes, as well as the reference map with sufficient density was required to ensure the precise identification of MQTLs.

### 4.3 Homology‑based CGs detected within MQTL regions

A total of 114 putative critical CGs within MQTL regions were identified based on significant gene expression in the spike which could influence the development of SL in wheat. It was hard to overstate the importance of considering the close evolutionary relationship between wheat and the model crop rice, so homology‑based CGs particularly showing higher expression in the spike are considered more important (Saini, Srivastava et al., 2022; Yang et al., 2021). For example, *TraesCS2A02G099400* of MQTL‐2A.1, encoding bZIP transcription factor, was highly expressed in the spike at the two nodes detectable stage. In contrast, the orthologues of this gene in rice, *OsbZIP62/OsFD7*, as reported in the previous study, were expressed preferentially in the shoot apical meristem and during early panicle developmental stages, playing a dual role in panicle development in rice (Kaur et al., 2021). The putative CGs encoding eEF1A‐like proteins, including *TraesCS2A02G444100* and *TraesCS2D02G443300*, were identified in the MQTL‐2A.3 and MQTL‐2D.2 regions which were specifically expressed at the spike ear emergence stage. In rice, the orthologues of these two genes *SPL33* were confirmed to show the highest expression in the spike and leaf, and the number of tillers, plant height, SL, and TGW of the *spl33* mutant decreased significantly (S. Wang, Lei et al., 2017). The MQTL‐2A.3 (*TraesCS2A02G407600*) and MQTL‐2D.4 (*TraesCS2D02G404700*) carried homologues of rice genes *SMG2*/*OsMKKK10* showing highest expression at the spike ear emergence stage. The suppression of mitogen‐activated protein kinase genes *OsMPK6*, *OsMKK4*, and *OsMKKK10* resulted in denser panicles and smaller grains (T. Guo, Chen et al., 2018). Relative to the wild type, the SL of *smg2*‐*1* became shorter due to the reduced length of the spike rachis (R. Xu et al., 2018). *TraesCS2A02G423500* and *TraesCS2D02G421200*, located within MQTL‐2A.3 and MQTL‐2D.4, encoding UAP1 protein, were also specifically expressed at the spike ear emergence stage. The homologous gene *SPL29* in rice was confirmed to encode the UAP1 protein. In the *spl29* mutant, plant height, SL, total grain number, grain filling rate, fruiting efficiency, and TGW were reduced compared to those of the wild type (Z. Wang et al., 2015). *TraesCS2D02G407700* of MQTL‐2D.4, encoding glutathione peroxidase, was strongly expressed at the spike flag leaf stage, while the mutant plant of its homologous gene *OsGPX5* showed a shorter adult plant stage, lower panicle length, and lower TGW compared to the WT (X. Wang, Fang et al., 2017). *TraesCS3B02G368300* of MQTL‐3B.1 was highly expressed at the spike two nodes detectable stage. In rice, the analysis of the expression of its homologous gene *OsbZIP09* and its encoded protein sequence and structure indicated that OsbZIP09 was a typical bZIP transcription factor containing a conserved bZIP domain (Zhu et al., 2021). There was no difference in plant height, the number of tillers per plant, number of grains per spike, TGW, and grain width of the mutant compared with the wild type; only SL and grain length increased to a certain extent (Brambilla et al., 2017; C. Wang et al., 2021). The plant height, SL, grain length, grain width, TGW, and yield per plant of the *OsPP2C09* overexpressed line increased at the maturity stage, while the deletion line showed the opposite (Y. Miao et al., 2020). In the present study, the homologous gene *TraesCS3B02G394600* in wheat was strongly expressed at the spike two nodes detectable stage, which was detected in the MQTL‐3B.2. *TraesCS4A02G099100* of MQTL‐4A.1 was highly expressed at the spike ear emergence stage, and its homologous gene *BC25* encodes a UDP‐glucuronic acid decarboxylase involved in cellulose synthesis. The mutant *bc25* showed shorter panicle length and lacked secondary branches (S. Xu et al., 2023). *TraesCS5B02G296600* and *TraesCS5D02G304400* exhibited significant splendid expression at the spike ear emergence stage, while the mutant of its homologous gene *OsFTL4* was reported in the early study which illustrated that the *osftl4* knockout mutation led to a reduction in plant height, panicle length, and grain number per panicle in rice, consequently reducing grain yield (Gu et al., 2022). All the CGs showing high expression in the spike were located in the MQTL regions identified in the present study and their homologous gene in rice has been characterized as affecting SL, indicating the feasibility of screening key CGs based on interspecific homology analysis.

### 4.4 Potential application of novel locus *TaPP2C‐3B* molecular marker in selection in wheat

The process of high‐yield wheat breeding involved the continuous accumulation of superior haplotypes of genes related to different yield traits (Y. Zhang, Li et al., 2021). SNP molecular markers based on KASP technology have significant advantages over other markers (Rasheed et al., 2016; Steele et al., 2018). In the recent study, many functional markers for essential agronomic traits in wheat were documented for variety development and germplasm enhancement through MAS, including *TaSPP*‐*5Aa* for increased sucrose content, TGW, and grain length (Jing et al., 2022); *TaGS5*‐*3A*‐*T* for larger grain size and TGW (L. Ma et al., 2016); *TaPPH*‐*7A*‐*1* (A) for higher TGW and chlorophyll content (H. Wang et al., 2019); *TaTAP46*‐*5A Hap1* for higher TGW and larger kernel size (Y. Zhang, Li et al., 2021); *TaTPP*‐*7A Hap1* for higher TGW and grain length (H. Liu, Si et al., 2023); and so on. In this study, a KASP marker was developed to distinguish between the two haplotypes of *TaPP2C‐3B*, and the results of the association analysis found that wheat varieties carrying *TaPP2C*‐*3B*‐*Hap II* showed significantly higher SL than those with *TaPP2C*‐*3B*‐*Hap I* (Figure 8). Although the dominant haplotype *TaPP2C*‐*3B*‐*Hap II* was not positively selected in wheat production regions in China, there was a significant increase in *TaPP2C*‐*3B*‐*Hap II* from the 1970s (0%) to post‐2010 (52.94%). Thus, there is significant potential for improving SL by selecting superior allelic variation *TaPP2C*‐*3B*‐*Hap II* in the process of high‐yield wheat breeding.

## 5 CONCLUSIONS

In conclusion, we succeeded in deciphering key genomic regions and high‐confidence CGs associated with SL in wheat. An improved MQTL analysis strategy was used to contribute to the identification of 48 stable and robust MQTLs; 11 of these were confirmed as core MQTLs. A total of 2240 CGs underlying 48 MQTLs were identified, including 58 CGs with homology in rice and 114 CGs with more than 2 TPM highly expressed in the spike by in silico gene expression analysis, of which the key gene for SL *TaPP2C*‐*3B* was performed for the further haplotype analysis. A functional KASP molecular marker for *TaPP2C*‐*3B* was developed based on an SNP at 272 bp (A/G). Association analysis demonstrated that two haplotypes were significantly associated with the SL in wheat, of which *TaPP2C*‐*3B*‐*Hap II* was a dominant haplotype with higher SL than those of *TaPP2C*‐*3B*‐*Hap I*. The *TaPP2C*‐*3B*‐*Hap II* showed positive selection in the process of wheat breeding from the 1990s to post‐2010. Further study may focus on the validation of the robustness and reliability of the results, such as through biological experiments, transgenics, and functional genomics techniques.

## AUTHOR CONTRIBUTIONS

**Changgang Yang**: Formal analysis; investigation; validation; visualization; writing—original draft. **Xueting Zhang**: Data curation; resources; software. **Shihong Wang**: Data curation; resources; software. **Na Liu**: Conceptualization; funding acquisition; project administration; writing—review and editing.

## CONFLICT OF INTEREST STATEMENT

The authors declare no conflicts of interest.

## Supporting information


**Table S1** Details of previous QTL studies used for Meta‐QTL analysis.
**Table S2** Description of 48 MQTLs identified for spike length.
**Table S3** The information of 2,240 genes identified in 48 MQTL regions.
**Table S4** The information of the wheat diversity panel and their genotypes of *TaPP2C*‐*3B*.
**Table S5** The information of the wheat diversity panel and their genotypes distribution of *TaPP2C*‐*3B* alleles.
**Table S6** The information of the wheat diversity panel and their genotypes of *TaPP2C*‐*3B* alleles in the different decades.

## Data Availability

The relevant data and additional information are available in the supplementary files.
